# Nuclear receptor corepressor 1 deficiency exacerbates asthma by modulating macrophage polarization

**DOI:** 10.1038/s41420-023-01724-3

**Published:** 2023-11-29

**Authors:** Chenchen Hou, Lifeng Yan, Ke Sun, Tianyu Zhou, Yuxin Zou, Weining Xiong, Sheng-Zhong Duan

**Affiliations:** 1 Department of Respiratory and Critical Care Medicine, Shanghai Ninth People’s Hospital, Shanghai Jiao Tong University School of Medicine, Shanghai, 200011 China; 2 Division of Nephrology, Huashan Hospital, Fudan University, Shanghai, 200031 China; 3 Shanghai Key Laboratory of Tissue Engineering, Shanghai Ninth People’s Hospital, Shanghai Jiao Tong University School of Medicine, Shanghai, 200011 China; 4 Laboratory of Oral Microbiota and Systemic Diseases, Shanghai Ninth People’s Hospital, College of Stomatology, Shanghai Jiao Tong University School of Medicine, Shanghai, 200011 China; 5 National Center for Stomatology, National Clinical Research Center for Oral Diseases, Shanghai Key Laboratory of Stomatology, Shanghai, 200011 China

**Keywords:** Experimental models of disease, Respiratory tract diseases

## Abstract

Macrophage polarization plays an important role in asthma. Nuclear receptor corepressor 1 (NCOR1) plays an important role in metabolic and cardiovascular diseases by regulating the function of macrophages. The aim of this research was to examine the role and mechanism of macrophage NCOR1 in the development of asthma. We used ovalbumin (OVA) to induce macrophage NCOR1-deficient mice for asthma formation. Our results revealed that macrophage NCOR1 deficiency markedly enhanced allergic airway inflammation. In addition, NCOR1 deficiency in macrophages was found to enhance M2 polarization. Mechanistic studies suggested that NCOR1 promoted macrophage polarization by interacting with PPARγ, contributing to the pathogenesis of asthma. In conclusion, macrophage NCOR1 deficiency promoted the regulation of M2 programming by enhancing PPARγ expression to exacerbate asthma. Macrophage NCOR1 might be a potential target for the treatment of asthma.

## Introduction

Asthma is characterized by chronic airway inflammation and reversible airway obstruction affecting ~334 million people worldwide [1]. Over the past few decades, the incidence of asthma has increased, resulting in a huge global health and economic burden [2]. Clinical medications for asthma, such as inhaled corticosteroids, do not change the natural history of asthma [3]. Therefore, more in-depth research on asthma is imperative.

Although the pathogenesis of asthma is still poorly understood, studies have indicated that genetic, environmental, infectious, and nutritional factors impact the emergence of asthma [4]. Notably, most patients with asthma have the hallmark of Th2 inflammation, which is associated with certain cytokines (IL-2, IL-5, and IL-14) and inflammatory cells (eosinophils, mast cells and macrophages) [5, 6]. Among them, macrophages, as the most enriched immune cells in the lung, show extremely high plasticity according to changes in the lung microenvironment. In general, macrophages can differentiate into the M1 phenotype (classically activated macrophages) when stimulated by interferon-γ (IFN-γ) or tumor necrosis factor-α (TNF-α) [7]. On the other hand, macrophages can differentiate toward the M2 phenotype (alternatively activated macrophages) after IL-4 or IL-13 treatment [8, 9]. In asthma, M2 macrophages infiltrate the airways of patients during disease progression and mediate type 2 immune responses [10]. At the same time, Th2-associated factors can further exacerbate macrophage differentiation toward the M2 phenotype, leading to a vicious cycle of asthma [11–13]. However, the exact mechanism of macrophage differentiation in asthma is still under investigation.

Nuclear receptor corepressor 1 (NCOR1) is a central regulator of transcription that inhibits the transcriptional activity of transcription factors by binding histone deacetylases to their corresponding sites [14]. These transcription factors include peroxisome proliferator-activated receptor (PPAR), estrogen-related receptors, and liver X receptor β (LXRβ) [15–17]. For example, NCOR1 interacts with LXRβ in microglia, and knockdown of NCOR1 in macrophages protects against ischemic stroke [18]. Deficiency of Ncor1 in macrophages exacerbates periodontitis by regulating Cebpα transcription through PPARγ, promoting osteoclastogenesis and neutrophil accumulation in mice with experimental periodontitis [19]. In a previous report, macrophage NCOR1 has been shown to prevent atherosclerosis via activation of PPARγ [15]. It has also been reported that M1-related genes are downregulated, while the expression of M2-type genes is upregulated in macrophage NCOR1 knockout (MNKO) mice [20]. Thus, macrophage NCOR1 may play a key role in asthma.

Therefore, we intended to investigate the involvement of macrophage NCOR1 in asthma. MNKO mice were used to investigate the effect of macrophage NCOR1 deletion on inflammation and macrophage differentiation in asthma. Finally, we investigated the cellular mechanism by which NCOR1 regulates macrophage differentiation in vitro.

## Results

### Macrophage NCOR1 deficiency exacerbates asthma in mice

**Fig. 1:**
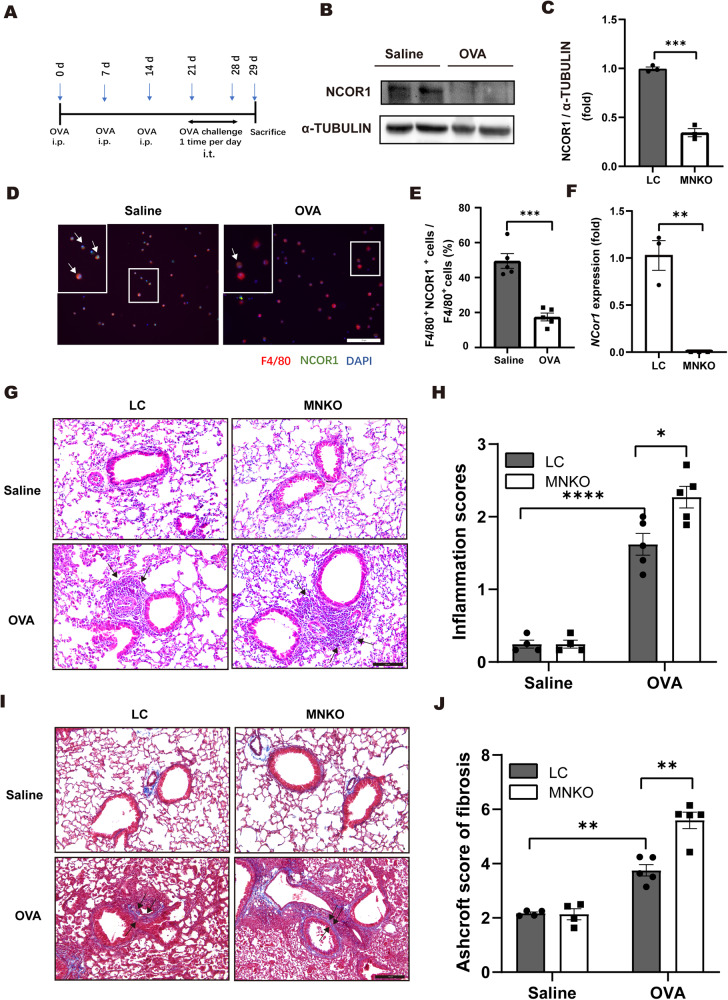
Macrophage NCOR1 deficiency exacerbates asthma in mice. **A** Schematic illustration of the experimental protocol for the mouse asthma model. OVA ovalbumin, i.p. intraperitoneal injection, i.t. intranasal injection. **B** Western blotting analysis of NCOR1 in mouse lung tissues. α-Tubulin was used as a loading control. **C** Quantification of western blotting exemplified in (**B**). **D** Representative immunofluorescence staining of F4/80 and NCOR1 in alveolar lavage fluid from control mice and allergic mice. The arrows point to F4/80/NCOR1 bi-positive cells. Scale bar: 50 µm. **E** Quantification of F4/80/NCOR1 double-positive cells. *n* = 5:5. **F** RT-QPCR analysis of *Ncor1* gene expression in BMDMs isolated from LC and MNKO mice. *n* = 3:3. **G** Hematoxylin and eosin staining. Scale bar: 100 μm. **H** Quantitative mean score of inflammation. *n* = 4:4:5:5. **I** Masson’s trichrome staining. Scale bar: 100 μm. **J** Quantitative mean score of fibrosis formation inflammation. *n* = 4:4:5:5. *p < 0.05, **p < 0.01, ***p < 0.001, ****p < 0.0001. BMDMs bone marrow-derived macrophages, LC littermate control, MNKO macrophage Ncor1 knockout.

In a mouse model of asthma induced by OVA peptides (Fig. 1A), the protein level of NCOR1 was significantly decreased in the lung tissues of asthmatic mice compared with control mice (Fig. 1B, C). Moreover, immunofluorescence staining illustrated that NCOR1 was markedly decreased in macrophages in the bronchoalveolar lavage fluid (BALF) of asthmatic mice (Fig. 1D, E). These results indicated an essential role of macrophage NCOR1 in the pathogenesis of asthma. To further investigate the role of macrophage NCOR1 during allergic airway inflammation, we generated MNKO mice by using the cre-lox system. RT-QPCR showed that NCOR1 mRNA levels were almost undetectable in bone marrow-derived macrophages (BMDMs) isolated from MNKO mice than LC mice (Fig. 1F) [20]. Hematoxylin and eosin (HE) staining showed that OVA-induced infiltration of inflammatory cells around the bronchovascular bundle was significantly higher in MNKO mice than in LC mice (Fig. 1G, H). Similarly, Masson’s trichrome staining demonstrated that OVA-induced peribronchial collagen deposition was significantly higher in MNKO mice (Fig. 1I, J).

**Fig. 2:**
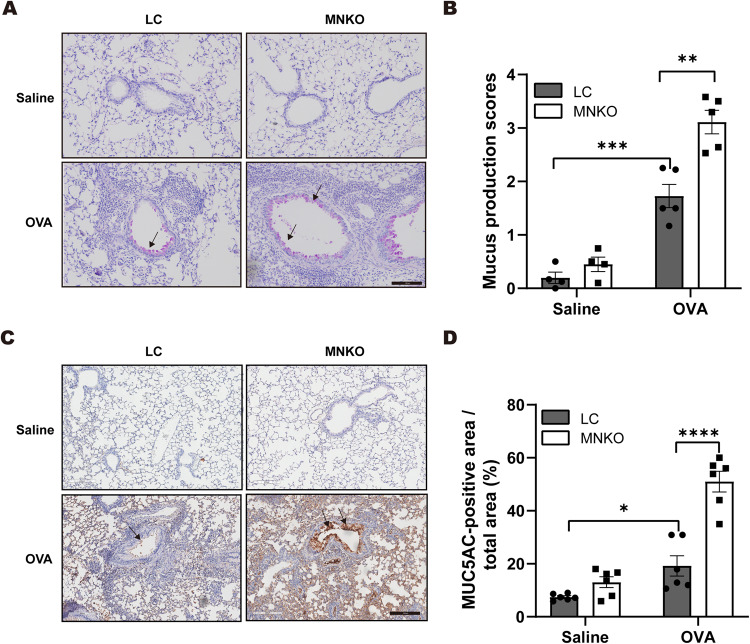
Macrophage NCOR1 deficiency exacerbates airway mucus secretion. **A** Periodic acid–Schiff (PAS) staining. Scale bar: 100 μm. **B** Quantitative mean score of mucus production. *n* = 4:4:5:5. **C** Representative immunohistochemical staining of MUC5AC expression in mouse lung sections. **D** Quantification of MUC5AC-positive areas as a percentage of total lung area. Scale bar: 200 µm. *n* = 6:6:6:6. *p < 0.05, **p < 0.01, ***p < 0.001, ****p < 0.0001.

We further used periodic acid–Schiff staining (PAS) to examine mucus production. Severe goblet cell hyperplasia and mucus hypersecretion were observed in OVA-challenged mice, and OVA-challenged MNKO mice had a significantly more severe phenotype compared with LC mice (Fig. 2A, B). In addition, we measured the mucus integral component MUC5AC by immunohistochemistry. Consistently, immunohistochemistry analysis demonstrated that asthmatic MNKO mice had a remarkable increase in the number of cells expressing MUC5AC compared with asthmatic LC mice, suggesting improved goblet-cell metaplasia after asthma (Fig. 2C, D). Collectively, these data suggested that macrophage NCOR1 deficiency exacerbated asthma in mice.

### Macrophage NCOR1 deficiency exacerbates Th2 inflammation in mouse asthma

**Fig. 3:**
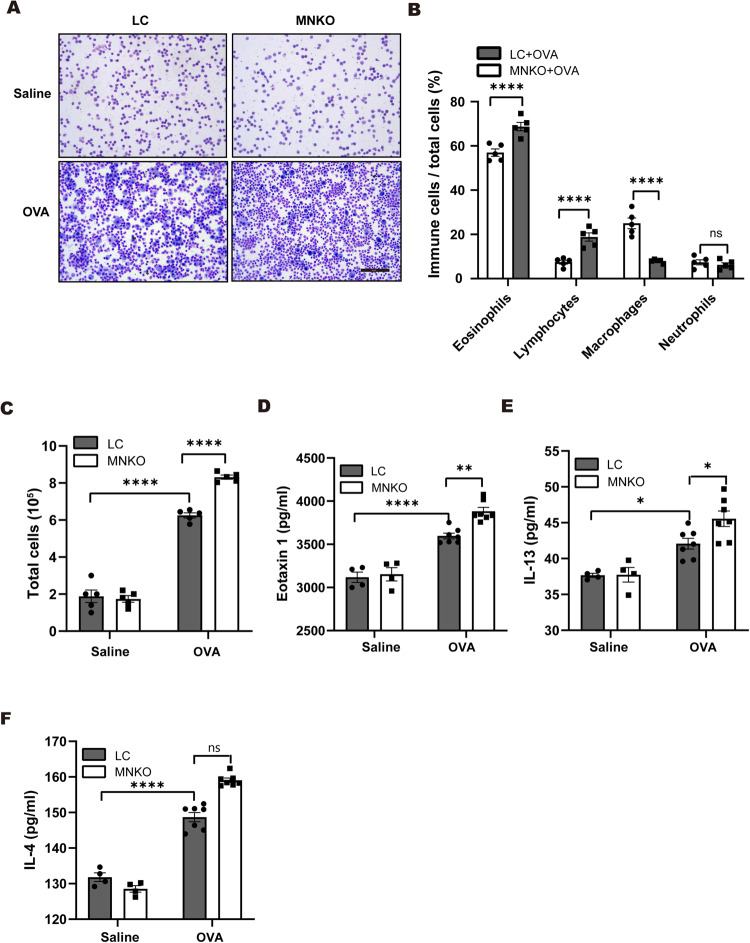
Macrophage NCOR1 deficiency exacerbates Th2 inflammation in mouse asthma. **A** Giemsa staining of mouse BALF cells. Scale bar: 50 μm. **B** Analysis of percentages of eosinophils, lymphocytes, macrophages and neutrophils in mouse total BALF cells. *n* = 5:5. **C** Total number of inflammatory cells in BALF samples. **D**–**F** ELISA detecting eotaxin 1, IL-13 and IL-4 secretion levels in mouse BALF supernatants. *n* = 4:4:7:7. *p < 0.05, **p < 0.01, ****p < 0.0001. BALF bronchoalveolar lavage fluid.

Next, we compared inflammatory cell subtypes and cytokines in the mouse BALF. Giemsa staining demonstrated that OVA induced significantly more eosinophils and lymphocytes and fewer macrophages in the BALF of MNKO mice than in that of LC mice (Fig. 3A, B). For the reason for the decrease in macrophages in BALF, we believed that NCOR1 deficiency in macrophages may affect macrophage proliferation [20]. More importantly, OVA-challenged MNKO mice showed significantly increased BALF total cells compared with OVA-challenged LC mice (Fig. 3C). Analysis of cytokines in BALF by ELISA revealed significantly higher levels of Th2 cytokines such as eotaxin1 and IL-13 in asthmatic MNKO mice than in asthmatic LC mice (Fig. 3D, E). The level of IL-4, another Th2 cytokine, showed an increasing trend in the BALF of asthmatic MNKO mice (Fig. 3F). Collectively, these results demonstrated that macrophage NCOR1 deficiency exacerbated Th2 cytokine production in the progression of asthma.

### Macrophage NCOR1 deficiency enhances M2 polarization

**Fig. 4:**
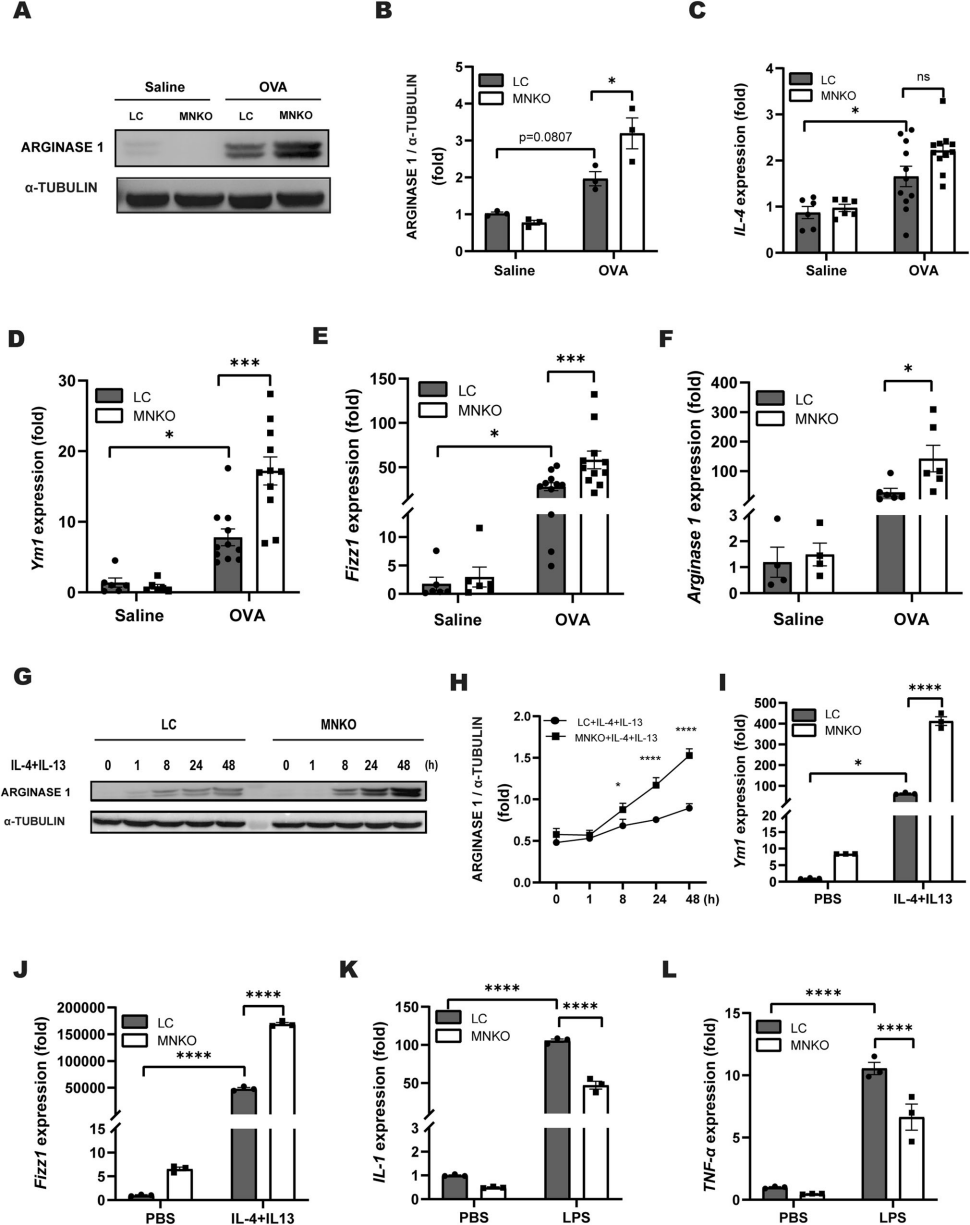
Macrophage NCOR1 deficiency enhances M2 polarization. **A** Western blotting analysis of Arginase 1 in mouse lung tissues. α-Tubulin was used as a loading control. **B** Quantification of western blotting exemplified in (**A**). **C**–**E** RT-QPCR analysis of *IL-4, Ym1* and *Fizz1* in lung tissues. *n* = 6:6:11:11. **F** RT-QPCR analysis of *Arginase 1* in lung tissues. *n* = 4:4:6:6. **G** Western blotting analysis of Arginase 1 in BMDMs after IL-4 and IL-13 stimulation. α-Tubulin was used as a loading control. **H** Quantification of western blotting exemplified in (**G**). **I**–**L** RT-QPCR analysis of *Ym1*, *Fizz1, IL-1* and *TNF-α* in BMDMs. *n* = 3:3:3:3. n.s not significant. *p < 0.05, ***p < 0.001, ****p < 0.0001. BMDMs bone marrow-derived macrophages.

Importantly, M2 macrophages play an important role in allergic asthma [21]. Indeed, the protein level of Arginase1, a signature for M2 macrophages, was increased in lungs from OVA-challenged LC mice compared with saline-treated LC mice (Fig. 4A, B). Therefore, we examined the effect of NCOR1 on macrophage M2 activation. Western blotting showed that the protein level of Arginase 1 was dramatically increased in asthmatic MNKO mice compared with asthmatic LC mice (Fig. 4A, B). Likewise, RT-QPCR analysis of IL-4, Ym1, Fizz1 and Arginase 1 showed consistent results (Fig. 4C–F). These results suggested that macrophage NCOR1 deficiency improved the induction of M2 macrophages. To evaluate the effect of MNKO on macrophage differentiation, we treated LC and MNKO BMDMs with IL-4 and IL-13 or LPS. The protein level of Arginase 1 was significantly increased in MNKO BMDMs compared with LC BMDMs after IL-4 and IL-13 stimulation (Fig. 4G, H), and similar results were obtained by RT-QPCR analysis of YM1 and Fizz1 (Fig. 4I, J). In addition, we also observed the effect of MNKO on M1 differentiation by stimulating BMDMs with LPS. RT-QPCR analysis revealed that after adding LPS, NCOR1 knockout in macrophages strongly inhibited LPS‐induced expression of M1 macrophage genes (Fig. 4K, L). Overall, these data suggested that macrophage NCOR1 deficiency enhanced M2 polarization and inhibited M1 polarization.

### PPARγ signaling mediates the effects of NCOR1 deficiency on M2 polarization

**Fig. 5:**
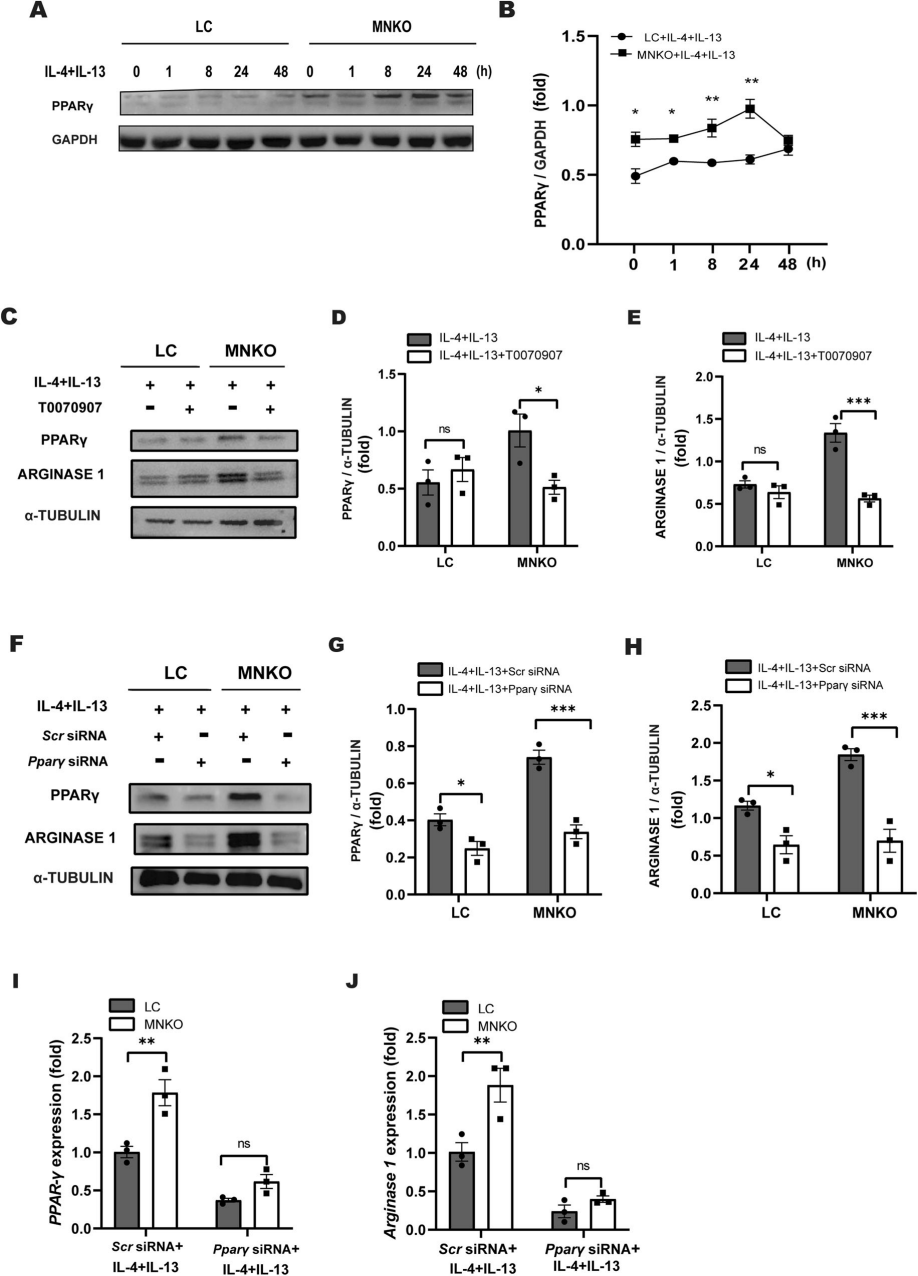
PPARγ signaling mediates the effects of NCOR1 deficiency on M2 polarization. **A** Western blotting analysis of PPARγ in BMDMs after IL-4 and IL-13 stimulation. **B** Quantification of western blotting results exemplified in (**A**). **C** Western blotting analysis of Arginase 1 and PPARγ in BMDMs treated with IL-4 and IL-13 with the PPARγ inhibitor T0070907. **D**, **E** Quantification of western blotting results exemplified in (**C**). **F** Western blotting analysis of Arginase 1 and PPARγ in BMDMs treated with IL-4 and IL-13 with Scr siRNA or Pparγ siRNA. **G**, **H** Quantification of western blotting results exemplified in (**F**). **I**, **J** RT-QPCR analysis of PPARγ and Arginase 1 in BMDMs treated with IL-4 and IL-13 with Scr siRNA or Pparγ siRNA. n = 3:3:3:3. n.s not significant. *p < 0.05, **p < 0.01, ***p < 0.001.

An intact IL-4/IL-13/PPARγ axis is critical for the maturation of macrophage M2 activation, and NCOR1 deficiency in macrophages leads to an alternative macrophage polarization phenotype upon PPARγ stimulation [15, 22]. Therefore, we used western blotting to detect the protein level of PPARγ in LC and MNKO BMDMs after IL-4 and IL-13 stimulation. Remarkably, IL-4 and IL-13 stimulation induced a significantly increased protein level of PPARγ in MNKO BMDMs compared with LC BMDMs in a time-dependent manner, indicating that NCOR1 deficiency enhanced PPARγ expression (Fig. 5A, B). To further demonstrate that PPARγ played a key role in macrophage M2 polarization in the absence of NCOR1, we treated LC and MNKO BMDMs with the PPARγ inhibitor T0070907 or *Pparγ* siRNA. Western blotting analysis showed that the protein level of PPARγ and Arginase 1 was significantly lower in MNKO BMDMs than in LC BMDMs after T0070907 treatment (Fig. 5C–E). Consistently, Western blotting and RT-QPCR analysis revealed that *Pparγ* siRNA efficiently repressed gene expression and the protein level of PPARγ in both LC and MNKO BMDMs (Fig. 5F, G, I). Accordingly, *Pparγ* siRNA notably decreased Arginase 1 gene expression and protein level in MNKO BMDMs compared with LC BMDMs (Fig. 5F, H, J). Collectively, these findings supported that macrophage NCOR1 deficiency promoted macrophage M2 polarization by enhancing PPARγ expression.

## Discussion

Although NCOR1 exerts a clear and important function in the pathogenesis of atherosclerosis and myocardial infarction [15, 20], it is unclear how macrophage NCOR1 affects asthma. In the present investigation, we demonstrated that NCOR1 deficiency in macrophages aggravated OVA-induced asthma. Mechanistically, NCOR1 deficiency promoted macrophage M2 polarization by enhancing PPARγ expression.

Macrophages are abundant and critical inflammatory cells in the respiratory tract and are an essential ingredient of the mononuclear phagocyte system [8]. In humans, mononuclear phagocytes are the main components of the interface between the lung and the external environment and play a key role in airway inflammation in asthma [23, 24]. Innate immune cells and lung epithelial cells are stimulated by allergens to create a range of cytokines that cause a disruption in the differentiation of resident lung macrophages, thereby exacerbating asthma [25]. In asthma with predominantly TH2-type inflammation, the dominant macrophages are M2 macrophages, which exhibit enhanced phagocytic activity but also produce proallergic factors such as YM1 and FIZZ1 [26–30]. M2 macrophages can activate TH2 cells, promote eosinophil infiltration into the lung, and contribute to airway inflammatory and remodeling responses. Previous studies have found that in severe asthma, especially in patients resistant to glucocorticoid therapy, macrophages are predominantly of the M1 phenotype [31]. It has been demonstrated that exosome membranes coated with EM-PLGA@Dnmt3aos^smart silencer^ biomaterials inhibit M0 to M2 polarization by targeting M2 macrophages for the treatment of asthma [32]. Furthermore, the addition of short-chain fatty acids butyrate and propionate to the diet relieves asthma by suppressing the polarization of M2 in the airways of mice [33]. Preliminary evidence suggests that targeted therapy against M2 macrophages may be part of the asthma treatment. Interestingly, our results suggested that M2 macrophage polarization was promoted in MNKO mice, suggesting that macrophage NCOR1 deficiency might exacerbate asthma by enhancing M0 to M2 polarization.

The next question to be addressed is to find the pathway by which NCOR1 in macrophages regulates M2 programming and thus exacerbates asthma. PPARγ, which has been extensively studied in the field of lipid metabolism and insulin signaling, also plays a crucial role in allergic diseases such as asthma [34]. In macrophages, STAT6/PPARγ signaling regulates the transcription of numerous genes, driving the M2 phenotype [35]. The significant increase in M2 macrophages in the airways of asthmatic patients compared to healthy controls also suggests a potential role for PPARγ in asthma and in allergic inflammation [25].

Previous studies have reported that NCOR1 is considered a tumor suppressor gene in lung adenocarcinoma, and its mutation is associated with poor prognosis of cancer, while high expression of NCOR1 is linked to better prognosis [36]. However, NCOR1 has not been studied in allergic diseases such as asthma. The role of NCOR1 in macrophages in atherosclerotic cardiovascular disease is complex and has been shown to have a protective function during atherosclerosis, but an increase in inflammatory cytokines is also observed in response to PPARγ agonist stimulation [15]. Furthermore, NCOR1 deficiency was found to inhibit proinflammatory factors released in the classical activation pathway of macrophages and to enhance the expression of M2-type anti-inflammatory genes in the context of myocardial infarction [20]. Consistently, the study showed that macrophage NCOR1 deficiency significantly enhanced the activity of PPARγ and the expression of proinflammatory cytokines in MNKO asthmatic mice. The results of the current study identified that NCOR1 depletion in macrophages disrupted the pulmonary immune system during the development of asthma.

However, our study has limitations. First, we conducted animal experiments with mice, which have a different pathological process from humans. Second, we established a mouse asthma model with ovalbumin, whose disease characteristics were consistent with hypereosinophilic asthma in the clinic. However, non-eosinophilic asthma also exists, and we have not studied in our experiments.

In summary, NCOR1 suppresses the inflammatory response during asthma development by regulating macrophage polarization. These results convincingly highlighted the pivotal biological functions of NCOR1 in the maintenance of lung homeostasis and the pathogenesis of asthma. Thus, it provided a potential novel strategy for designing clinical therapeutic strategies against asthma.

## Methods

### Animals

*Ncor1*^flox/flox^ mice on a C57BL/6J background were backcrossed to mice that have lysozyme M promoter-controlled Cre recombinase expression to create *Ncor1*^flox/flox^;*LysM*^cre^ (MNKO) mice and *Ncor1*^flox/flox^ (LC) mice [17]. Genotypes of all animals were confirmed. Eight-week-old male mice were utilized in the study. All experimental operations were authorized by the Institutional Review and Ethics Board of Shanghai Ninth People’s Hospital, Shanghai Jiao Tong University School of Medicine.

### Allergen-induced mouse asthma model

Asthma was induced in LC and MNKO mice as previously described [37, 38]. Briefly, on days 0, 7, and 14, mice received intraperitoneal injections of 100 g ovalbumin. Control mice received the same dosage of regular saline injection. Then, the sensitized mice were challenged on day 21 to day 28 with 50 μl of OVA (20 μg/μl) or saline under isoflurane anesthesia (Yipin, Hebei, China).

### BALF

BALF was gathered by intubating and washing the lungs with 0.8 ml of cold PBS. Afterwards, samples were centrifuged at 400 × *g* for 5 min. After being resuspended in 1 ml of PBS, the precipitated cells were enumerated. Finally, inflammatory cells were counted by Wright-Giemsa (Baso, Zhuhai, China) staining.

### Cell culture

As previously disclosed, mouse bone marrow-derived macrophages (BMDMs) were extracted and cultivated [20]. In summary, mouse femurs and tibias were used to collect bone marrow cells, which were then cultivated in RPMI 1640 (Thermo Fisher Scientific, Carlsbad, USA) complete medium containing 30 ng/ml macrophage colony-stimulating factor (M-CSF; Novoprotein, Shanghai, China). Interleukin (IL)-4 (10 ng/ml, Novoprotein, Shanghai, China) and IL-13 (10 ng/ml, Novoprotein, Shanghai, China) or LPS (10 ng/ml, Novoprotein, Shanghai, China) were used to treat macrophages for the indicated periods of time.

### siRNA

The sequences were as follows: *Pparγ* siRNA, 5′GCUCCAAGAAUA CCAAAGUTT3′ and *Scr* siRNA, 5′UUCUCCGAACGU GUCACGUTT3′. BMDMs were transfected with *Pparγ* siRNA or *Scr* siRNA using Lipofectamine 2000 (Thermo Fisher Scientific, Carlsbad, USA).

### ELISA

Mouse BALF was collected and centrifuged to extract the supernatant. The cytokines eotaxin 1, IL-13, IL-4 and TNF-α were measured by using ELISA kits (Mlbio, Shanghai, China). All ELISA experiments were performed according to the directions provided by the manufacturers.

### Histology

The lung was fixed with 4% paraformaldehyde for 1 min at 25 cm H2O pressure. Then, the lung was put in new 4% paraformaldehyde for 24 h, and sections of lung tissues were prepared (5 μm) and stained with Masson’s staining, periodic acid-Schiff, and hematoxylin and eosin.

### Immunohistochemistry

After 1 h of blocking at 37 °C, the sections were treated with mouse primary antibodies (Cell Signaling Technology, USA) at 4 °C overnight. The following day, secondary antibody (Proteintech, China) was incubated with tissue slices at 37 °C for 1 h. Afterwards, 3,3-diaminobiphenylpyridine (DAB) was used as a chromogenic agent, and hematoxylin was used to stain nuclei. Finally, ethanol and xylene were used for dehydration. The images were then observed by microscopy.

### Immunofluorescence

For immunocytochemical treatment, mouse alveolar lavage fluid cells were adhered to coverslips by shaking the slides. Coverslips were fixed in 4% paraformaldehyde for 10 min at 37 °C. Then, PBS blocking buffer was applied to the coverslips for 2 h at 37 °C, and then the primary antibody (Cell Signaling Technology, USA) was applied overnight at 4 °C. Secondary antibody (Thermo Fisher Scientific, USA) was used the next day for 1 h at 37 °C. Finally, the sections were stained with DAPI (Thermo Fisher Scientific, USA) for cell nuclei, and the fluorescence signal was captured by fluorescence microscopy.

### Quantitative RT-PCR

Using TRIzol (Thermo Fisher Scientific, USA) to extract gross RNA from lung samples or cells according to the product instructions, and synthetizing cDNA by using a reverse transcription box (Takara, Shiga, Japan). RT-QPCR products were assayed with SYBR Green mixture (Thermo Fisher Scientific, USA). GAPDH was used for normalization. The following primers were used: NCOR1,5′-CTG GTC TTT CAG CCA CCA TT-3′ and 5′-CCT TCA TTG GAT CCT CCA TC-3′; Arg1,5′-CAT TGG CTT GCG AGA CGT AGA C-3′ and 5′-GCT GAA GGT CTC TTC CAT CAC C-3′; Fizz1,5′-CAA GGA ACT TCT TGC CAA TCC AG-3′ and 5′-CCA AGA TCC ACA GGC AAA GCC A-3′; IL-1,5′-GCA ACT GTT CCT GAA CTC AAC T-3′ and 5′-ATC TTT TGG GGT CCG TCA ACT-3′; Ym1,5′-GGG CAT ACC TTT ATC CTG AG-3′ and 5′-CCA CTG AAG TCA TCC ATG TC-3′; TNF-α,5′-CCC TCA CAC TCA GAT CAT CTT CT-3′ and 5′-GCT ACG ACG TGG GCT ACA G-3′; IL-4,5′-CGC TGA ACA TCC TCA CAA CG-3′ and 5′-AGA ACA GGT CTT GCT TGC CA-3′; PPARγ,5′-CCC TTT ACC ACG GTT GAT TTC R-3′ and 5′-ACT TCA ATC GGA TGG TTC TTC G-3′ and Gapdh,5′-ATG TTC CAG TAT GAC TCC ACT CAC G-3′ and 5′-GAA GAC ACC AGT AGA CTC CAC GAC A-3′.

### Western blotting analysis

Lysis of lung tissues and cells with lysis buffer to extract total protein. SDS‒PAGE was used to isolate protein samples, which were then transmitted to PVDF membranes. Then, the membrane was probed with primary antibodies and incubated with secondary antibodies. Finally, chemiluminescence substrates (Thermo Fisher Scientific, USA) were used to visualize the reactive bands. The primary antibodies included NCOR1 (Cell Signaling Technology, USA), ARGINASE 1 (Proteintech, China), PPARγ (Proteintech, China), α-TUBULIN (Sigma Aldrich, USA), and GAPDH (Cell Signaling Technology, USA).

### Statistical analysis

All information was displayed as the mean ± SEM. GraphPad Prism 8.0 Program was utilized to perform the statistical analysis. In pairwise comparisons, Student’s *t* test was employed for analysis. Two-way ANOVA was used to analyze multiple comparisons. For all statistical comparisons, the significance level was established at 0.05.

### Supplementary information

Original Data File

## Data Availability

All data are available in the main text or the Supplementary Materials.
