# Supplementary material for: Nuclear receptor corepressor 1 deficiency exacerbates asthma by modulating macrophage polarization
Source: Cell Death Discov. 2023 Nov 29;9:429. doi: 10.1038/s41420-023-01724-3 (PMC10687133; doi:10.1038/s41420-023-01724-3)

Original WB

Supplementary File 1

Figure 1B

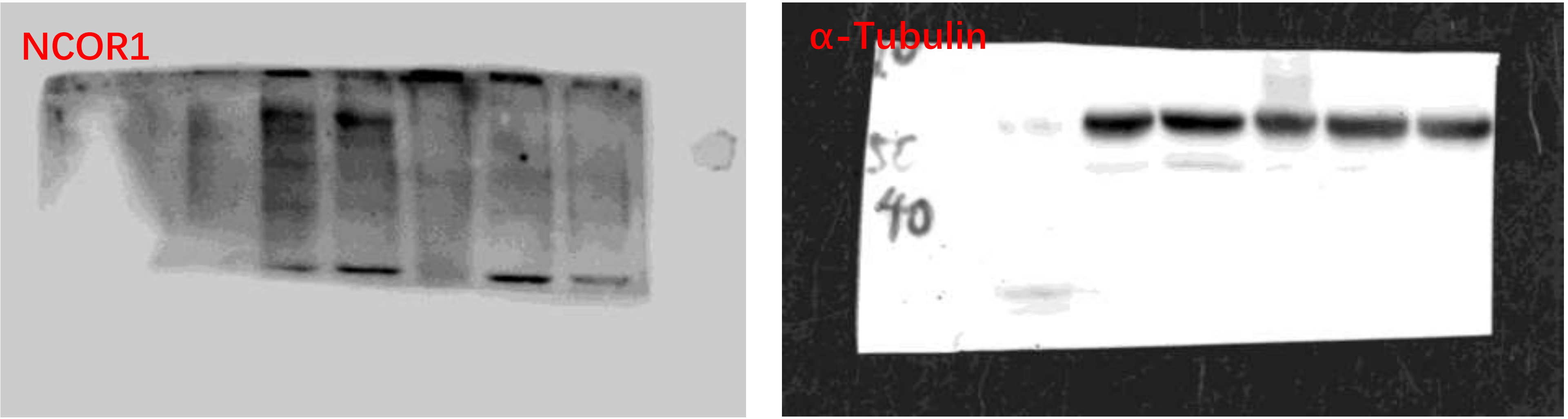

Supplementary File 2

Figure 4A

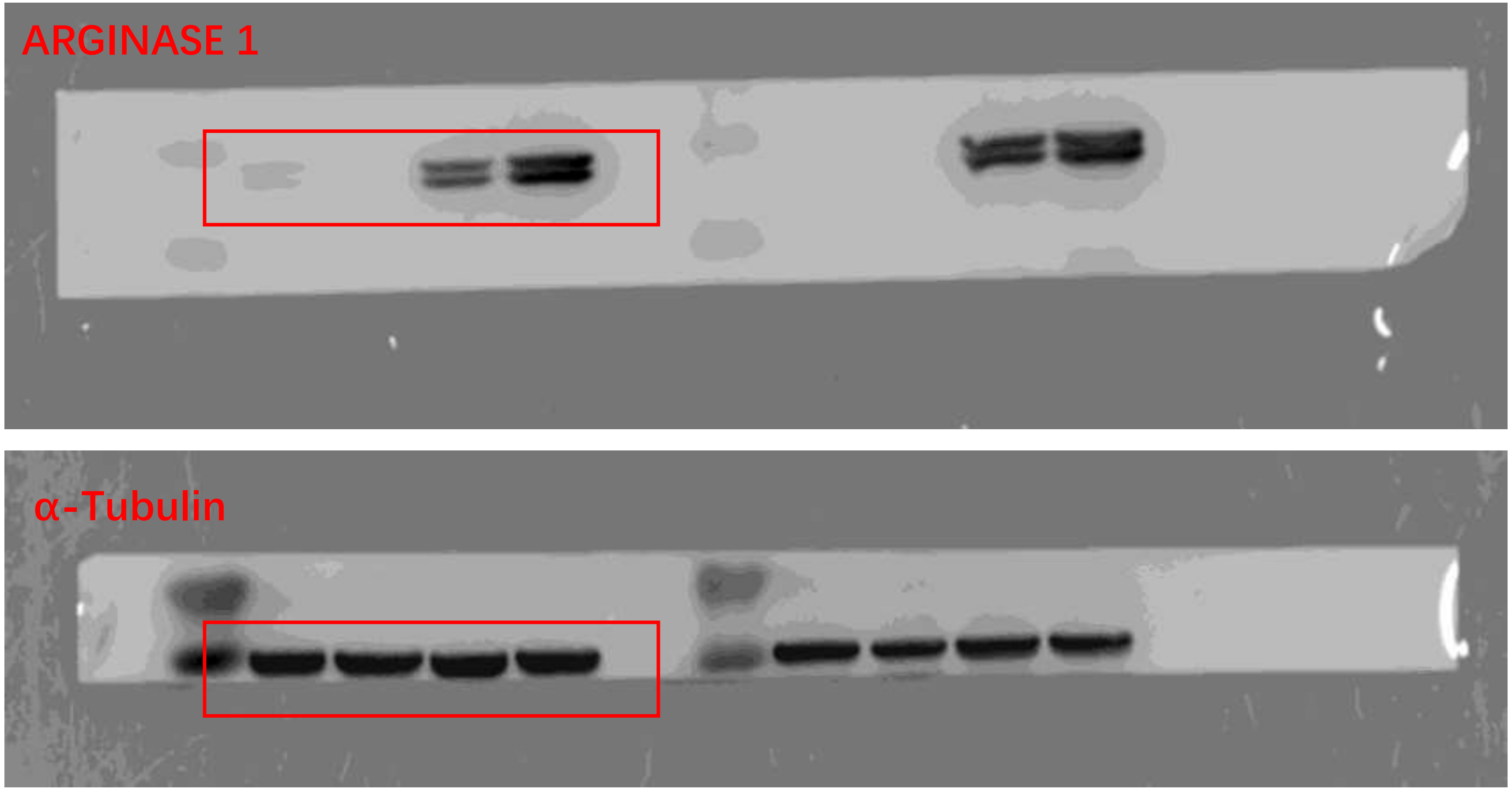

Supplementary File 3

Figure 4G

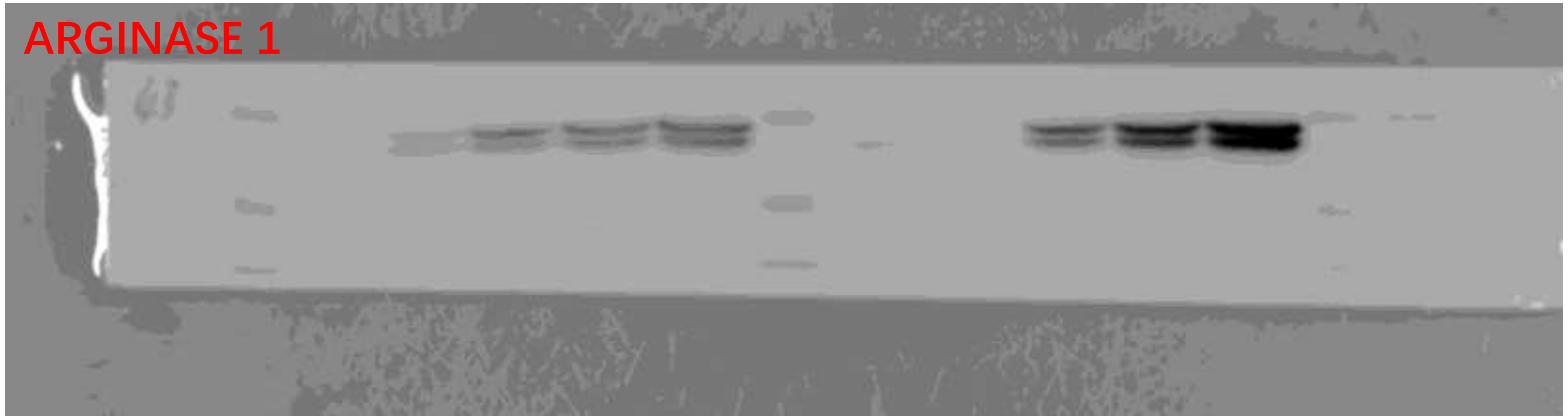

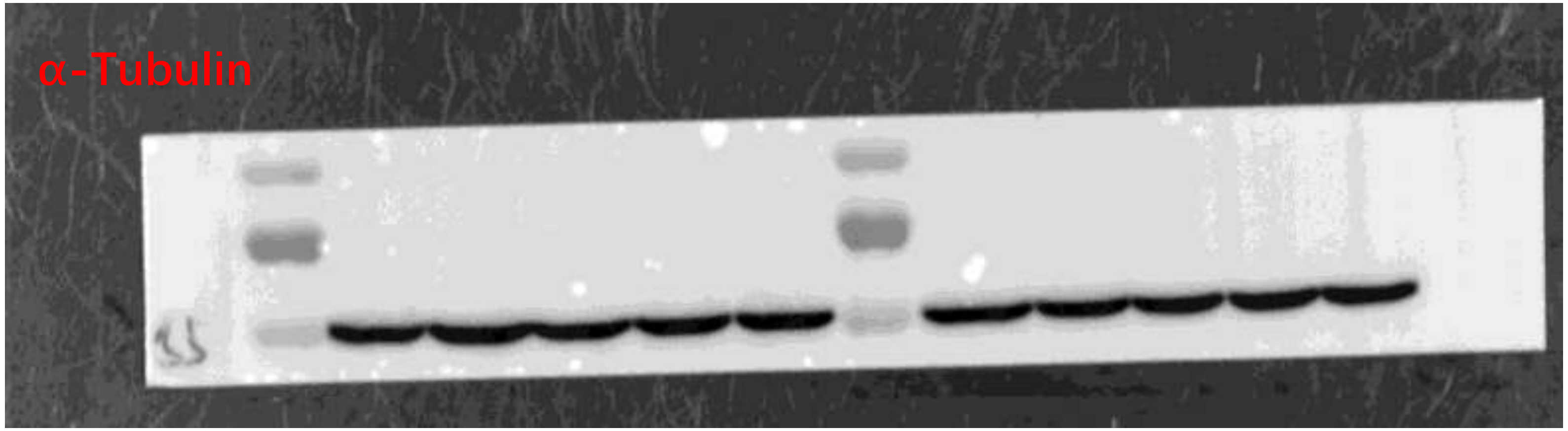

Supplementary File 4

Figure 5A

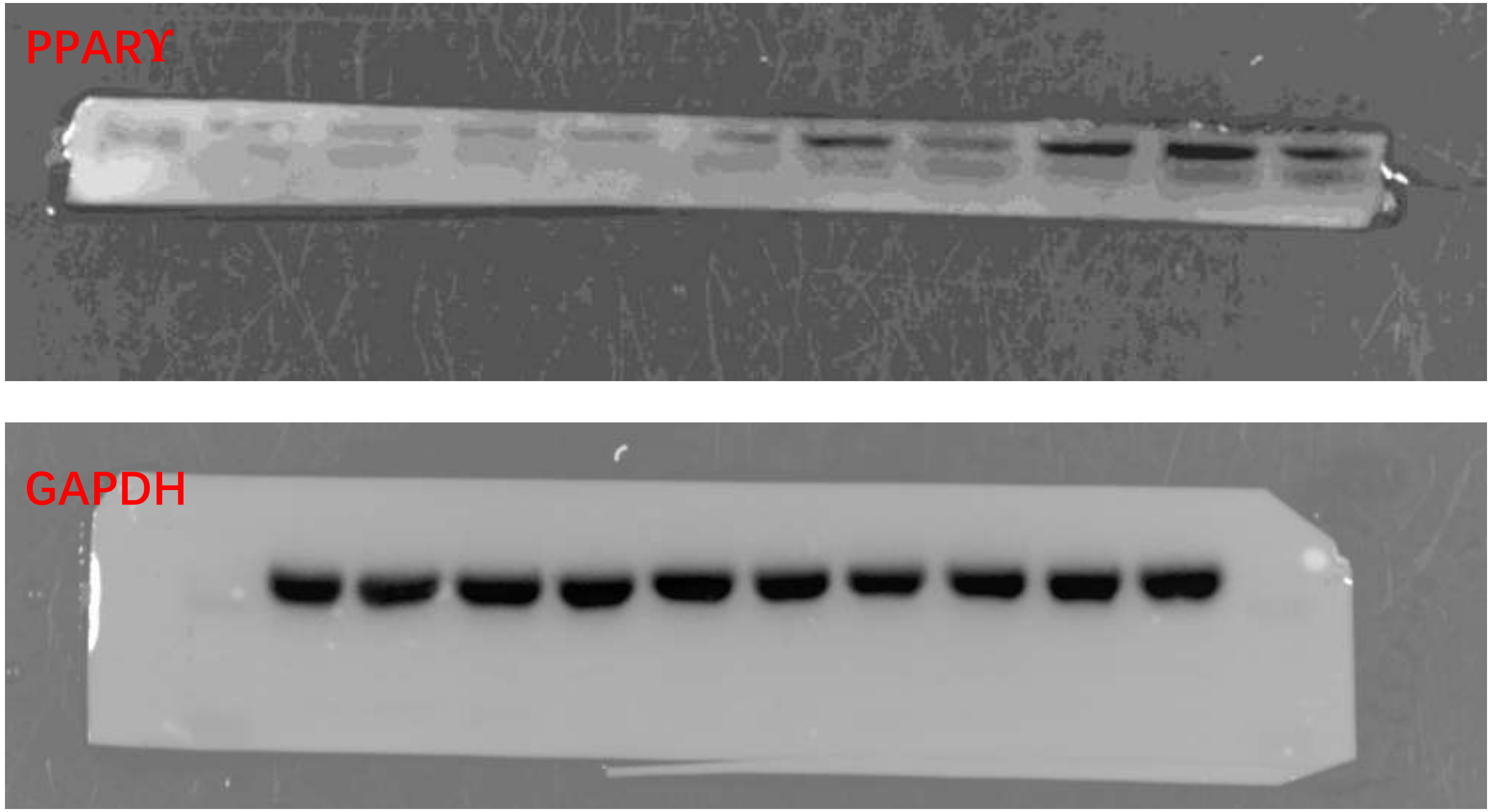

Supplementary File 4

Figure 5C

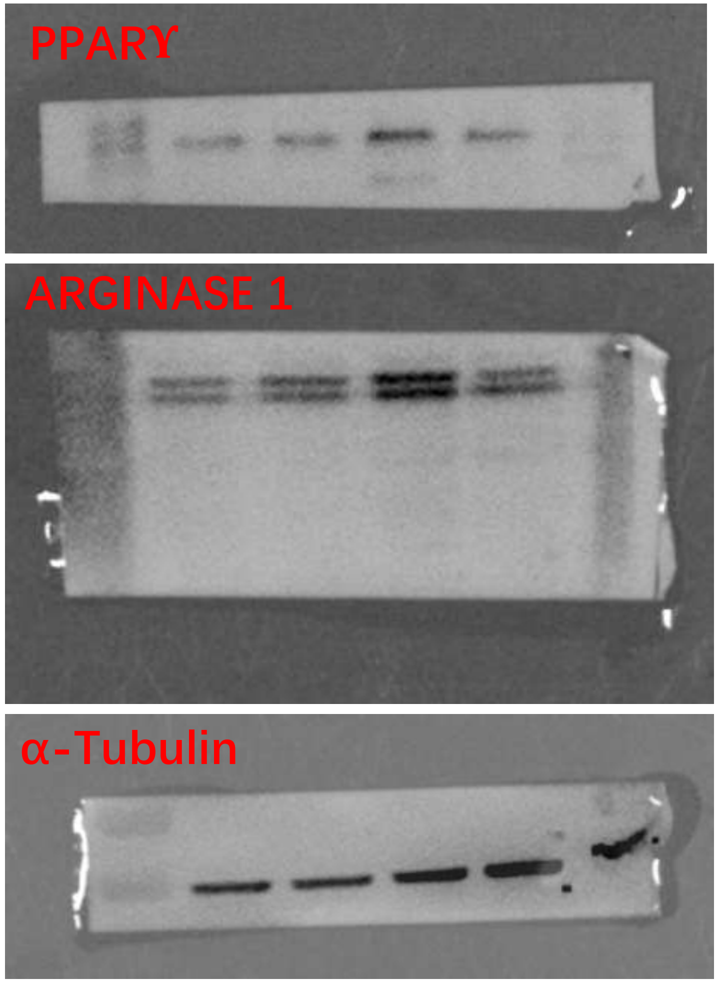

Supplementary File 4

Figure 5F

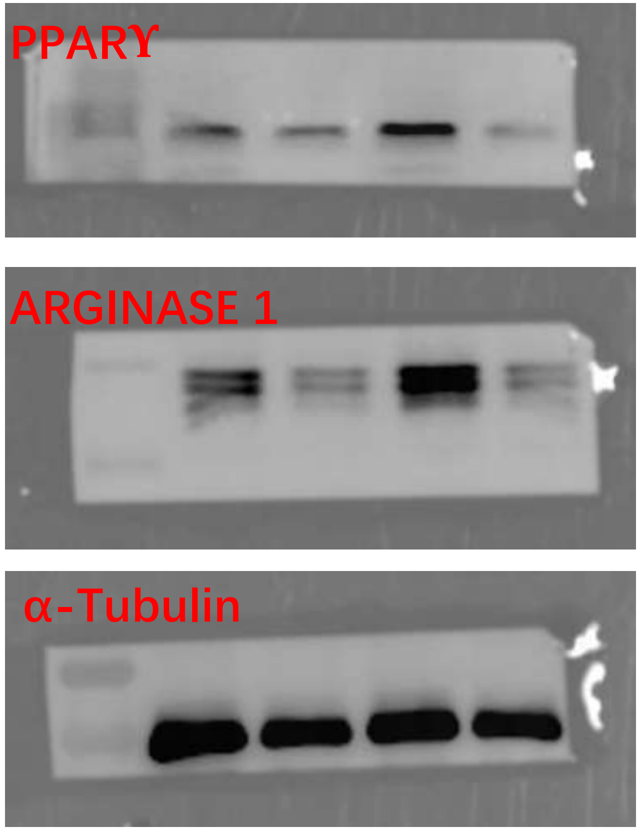

Supplement: Supplementary file 1 — Original Data File [file 41420_2023_1724_MOESM1_ESM.pdf]
